# Hospitalisation for venous thromboembolism in cancer patients and the general population: a population-based cohort study in Denmark, 1997–2006

**DOI:** 10.1038/sj.bjc.6605883

**Published:** 2010-09-14

**Authors:** D P Cronin-Fenton, F Søndergaard, L A Pedersen, J P Fryzek, K Cetin, J Acquavella, J A Baron, H T Sørensen

**Affiliations:** 1 Department of Clinical Epidemiology, Aarhus University Hospital, Olof Palmes Allè 43-45, Aarhus N 8200, Denmark; 2 Department of Epidemiology, Amgen, Thousand Oaks, CA, USA; 3 Departments of Community and Family Medicine and Medicine, Dartmouth Hitchcock Medical Center, Lebanon, NH, USA

**Keywords:** incidence rate, venous thromboembolism, epidemiology, treatment

## Abstract

**Background:**

Venous thromboembolism (VTE) frequently complicates cancer. Data on tumour-specific VTE predictors are limited, but may inform strategies to prevent thrombosis.

**Methods:**

We computed incidence rates (IRs) with 95% confidence intervals (CIs) for VTE hospitalisation in a cohort of cancer patients (*n*=57 591) and in a comparison general-population cohort (*n*=287 476) in Denmark. The subjects entered the study in 1997–2005, and the follow-up continued through 2006. Using Cox proportional-hazards regression, we estimated relative risks (RRs) for VTE predictors, while adjusting for comorbidity.

**Results:**

Throughout the follow-up, VTE IR was higher among the cancer patients (IR=8.0, 95% CI=7.6–8.5) than the general population (IR=4.7, 95% CI=4.3–5.1), particularly in the first year after cancer diagnosis (IR=15.0, 95% CI=13.8–16.2, *vs* IR=8.6, 95% CI=7.6–9.9). Incidence rates of VTE were highest in patients with pancreas (IR=40.9, 95% CI=29.5–56.7), brain (IR=17.7, 95% CI=11.3–27.8) or liver (IR=20.4, 95% CI=9.2–45.3) tumours, multiple myeloma (IR=22.6, 95% CI=15.4–33.2) and among patients with advanced-stage cancers (IR=27.7, 95% CI=24.0–32.0) or those who received chemotherapy or no/symptomatic treatment. The adjusted RR (aRR) for VTE was highest among patients with pancreas (aRR=16.3, 95% CI=8.1–32.6) or brain cancer (aRR=19.8 95% CI=7.1–55.2), multiple myeloma (aRR=46.1, 95% CI=13.1–162.0) and among patients receiving chemotherapy, either alone (aRR=18.5, 95% CI=11.9–28.7) or in combination treatments (aRR=16.2, 95% CI=12.0–21.7).

**Conclusions:**

Risk of VTE is higher among cancer patients than in the general population. Predictors of VTE include recency of cancer diagnosis, cancer site, stage and the type of cancer-directed treatment.

Since Trousseau's observation in 1865 (Trousseau, 1865), venous thromboembolism (VTE) has been widely documented as a serious complication of malignancy (Rickles and Levine, 2001; Prandoni *et al*, 2005; Blom *et al*, 2006b). Factors implicated include tumour-induced hypercoagulability; vascular injury caused by tumour, treatment or surgery; and, among bed-ridden cancer patients, venous stasis due to immobilisation (Gouin-Thibault *et al*, 2001; Prandoni *et al*, 2005; Zwicker *et al*, 2009).

The identification of factors associated with the incidence and clinical time-course of VTE in cancer patients compared with the general population is fundamental for further understanding of the association between cancer and VTE, and potentially prevent the occurrence of VTE. Risk factors for VTE include cancer type (adenocarcinomas of the viscera, brain and urogenital cancers); advanced stage; and cancer therapies, such as chemotherapy and surgery (Otten *et al*, 2004; Chew *et al*, 2006; Ogren *et al*, 2006; Stein *et al*, 2006; Khorana *et al*, 2007; Rodriguez *et al*, 2007). Although there is evidence that cancer patients have twice the risk of VTE compared with non-cancer patients undergoing the same surgical procedures (Rickles and Levine, 2001), few investigations have directly compared VTE incidence in cancer patients with cancer-free members of the general-population (Blom *et al*, 2006b; Heit *et al*, 2001; White *et al*, 2007). None of the previous studies were able to implement matching, which, in cohort studies, enables control of potential confounding at the design stage.

We took advantage of Danish population-based registries to conduct a study of predictors of VTE, including cancer site, stage, treatment and time since diagnosis, in cancer patients using a matched cohort design with prospectively collected data, a task which is prohibitively expensive in conventional epidemiological settings.

## Materials and methods

### Study population

We conducted this cohort study among individuals aged ⩾15 years residing in northern Denmark (1.8 million inhabitants). In Denmark, all medical records are tracked for individual patients using their civil personal registration number—a unique identifier encoding sex and date of birth—assigned to all Danish residents since 1968. Using the civil personal registration number, we linked data from the Danish National Registry of Patients (DNRP), the Danish Cancer Registry (DCR) and the Danish Civil Registration System (Andersen *et al*, 1999; Frank, 2000; Pedersen *et al*, 2006).

The DNRP has tracked acute non-psychiatric hospitalisations since 1977 and outpatient and emergency-room visits since 1995; diagnoses have been coded using the eighth revision of the International Classification of Diseases (ICD-8 (Sundhedsstyrelsen, 1986)) through 1993 and the tenth revision (ICD-10 (Sundhedsstyrelsen, 1993)), thereafter. Information is recorded immediately after discharge or outpatient visit and includes admission and discharge dates, and up to 20 diagnoses (Andersen *et al*, 1999). We obtained complete hospital history (including VTE) for the cancer and general-population cohorts and linked the resulting data set to records in the Civil Registration System, which tracks vital status and migration nationwide.

### Cancer cohort

From the DNRP, we identified individuals in the study area with a first cancer diagnosis, excluding non-melanoma skin cancer (ICD-10 codes: C00-C97.9) recorded between January 1, 1997 and December 31, 2005. We chose this period to ensure homogeneity of VTE diagnostic procedures (for example, ultrasound for deep vein thrombosis) for the included cancer patients (Lensing *et al*, 1989). The date of cancer diagnosis was that specified in the DNRP. We eliminated cases (∼6%) for which a hospital diagnosis did not correspond to an incident cancer recorded at the same site in the DCR. All Danish cancer cases are reportable to the DCR and recorded using the ICD-7 (seventh revision) since 1943 and ICD-O (oncology revision) since 1977. The DCR is over 95% complete and has almost 100% validity (Storm *et al*, 1997). For cancers diagnosed in 2004–2005, we included patients with cancers recorded in the DNRP only because DCR records were not available for this period.

Because VTE can indicate undiagnosed cancer (Baron *et al*, 1998; Sorensen *et al*, 1998), we excluded cancer patients diagnosed with VTE in the year before their cancer diagnosis (*n*=124) from all analyses.

### General-population cohort

We used the Civil Registration System to assemble a general-population comparison cohort (Frank, 2000). For each patient with cancer, we randomly selected five general-population members from a pool of individuals who were alive and free of cancer on the date of the matched person's cancer diagnosis as recorded in the DNRP (the index date), matched on birth year, sex and county of residence.

To maintain comparability of the cohorts, we also excluded from the pool of the general-population members available for matching persons who had been diagnosed with VTE in the year before the index date.

### Tumour predictors of VTE

In sub-analyses limited to cancer patients and their matched comparison group diagnosed while DCR records were available (<2004), we ascertained information on cancer site from the DCR. The DCR records data on cancer stage and treatment administered within 4 months of diagnosis (initial treatment). We classified cancer stage according to Tumour Node Metastasis stages I, II, III, IV and unknown. To examine VTE incidence by treatment and stage, we conducted a sub-analysis, including patients with records in the DCR and DNRP through 2003 and their matched members of the general-population, yielding a 6-year maximum follow-up.

### Comorbidity data

We used the DNRP to retrieve information on history of inpatient diagnoses of potential confounding diseases. We ascertained the following diagnoses: myocardial infarction (ICD-8:410; ICD-10:I21), congestive heart failure (ICD-8:427; ICD-10:I50.0), atherosclerosis and peripheral vascular disease (ICD-8:440; ICD-10:I73), chronic obstructive pulmonary disease (ICD-8:491; ICD-10:J44), inflammatory bowel disease (ICD-8:563; ICD-10:K50–K52), peptic ulcer disease (ICD-8:531–533; ICD-10:K27), liver disease (ICD-8:570–573; ICD-10:K70–K77), renal disease (ICD-8:400–404; ICD-10:I10–I15), diabetes (ICD-8:249 and 250; ICD-10:E10–E14), obesity (ICD-8:277; ICD-10:E66), pancreatitis (ICD-8:577.00–577.09; ICD-10:K85), alcoholism and alcoholism-related conditions (ICD-8:291–303; ICD-10:F10) and hypertension (ICD-8:400–404; ICD-10:I10–I15).

### VTE data

Individuals were followed-up from the cancer diagnosis/index date until an inpatient VTE diagnosis, death, emigration or 31 December 2006, whichever came first, or until cancer diagnosis for members of the general-population cohort, for 9 years maximum follow-up. We did not include individuals with an outpatient or emergency-room VTE diagnosis without a subsequent inpatient diagnosis, because such diagnoses were likely to represent coding errors (Severinsen *et al*, 2010). We used all diagnosis fields in the DNRP to identify VTE events that occurred after cancer diagnosis/index date and included pulmonary embolism (ICD-10: I26), phlebitis and thrombophlebitis (deep vein thrombosis or superficial thrombosis—ICD-10: I80) and other venous embolism and thrombosis (ICD-10: I81 and I82).

### Statistical analyses

We computed crude incidence rates (IRs) of hospitalisation for VTE as the number of cases per 1000 person-years (p-y) and associated 95% confidence intervals (CI) for the cancer and general-population cohorts. Among the cancer patients, we estimated VTE incidence by patient, tumour and treatment characteristics and by time since cancer diagnosis. Incidence rates were compared using the Poisson distribution; two-sided *P*-values <0.05 were considered statistically significant. We compared IRs of VTE between men and women for cancers that affect both men and women. To describe time to and absolute risk of VTE, we constructed Nelson–Aalen plots using product-limit methods (Ludbrook and Royse, 2008) illustrating cumulative incidence for VTE in select cancers.

We used Cox proportional-hazards regression to estimate the hazard ratio as a measure of the relative risk (RR) of VTE among cancer patients compared with the general-population, adjusting for comorbidity. For the analysis of time since diagnosis, additional adjustment for age and sex was done in the regression model to account for any age and sex imbalances potentially produced by differences in the cohort composition after the diagnosis/index date. We examined the RR of ‘provoked’ and ‘unprovoked’ VTE by stratifying our analyses by the receipt of surgery within 90 days before the VTE diagnosis (Glynn and Rosner, 2005).

In an analysis restricted to the cancer patients, we also computed RRs to assess the association between VTE risk and cancer site, stage and initial treatment, adjusting for age, sex, county and comorbidity using colon cancer as a reference group. Cox proportional-hazards regression was also used to examine whether any cancer site-related differences were explainable by stage and/or treatment.

## Results

### Descriptive data

We identified 57 591 incident cancer cases diagnosed between 1997 and 2005 and matched 287 476 individuals without cancer from the general-population (Table 1). Follow-up spanned 127 492 p-y for the cancer cohort (median: 1.23 p-y) and 1 087 946 p-y for the general-population cohort (median: 3.46 p-y). The most common cancer sites were the colorectum, lung and breast, each representing approximately 14% of all cancers. There were slightly more women than men in the study sample (52 versus 48%) and 69% of the sample were aged at least 60 years at cancer diagnosis/ index date.

### Incidence rate of hospitalisation for VTE

The overall IR of VTE in cancer patients was 8.0 cases per 1000 p-y (95% CI=7.6–8.5, Table 2). Incidence was highest during the first year following cancer diagnosis (15.0 cases per 1000 p-y, 95% CI=13.8–16.2), declining to 6.3 cases per 1000 p-y (95% CI=5.4–7.3) during the second year following cancer diagnosis and to 4.2 cases per 1000 p-y (95% CI=3.7–4.7) thereafter (Supplementary Table 2). For cancers that affect men and women, the rate of VTE in men (IR=10.0 cases per 1000 p-y, 95% CI=9.1–11.0) was very similar to that in women (IR=10.1 cases per 1,000 p-y, 95% CI=9.1–11.3), (*P*=0.99).

**Figure 1:**
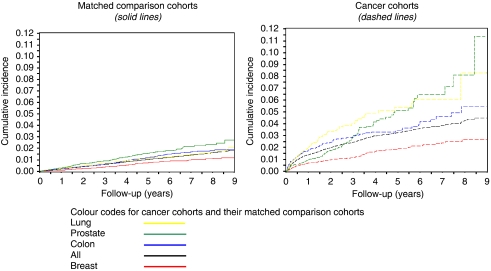
Cumulative incidence of hospitalisation for venous thromboembolism (VTE) in the cancer and general-population cohorts overall and for the four most common cancer types (Danish National Registry of Patients, 1997–2005).

The cumulative incidence of VTE after cancer diagnosis initially rose sharply, with a diminishing rate of increase over subsequent years (Figure 1). Overall, during the first year of follow-up, VTE was diagnosed in 1.4% of cancer patients and in 0.2% of the general-population cohort, and this difference varied by cancer site (e.g., 4.4% for pancreas and 0.7% for breast *vs* 0.3 and 0.1% in the general-population comparators for these cancers, respectively). VTE IRs were highest for patients with pancreas, liver, lung, ovary and brain cancers, and for multiple myeloma (Supplementary Table 2). Overall, the IRs of VTE were higher in the first year after the index date than in subsequent years. However, for some cancer sites (pancreas, liver and lung) the CIs associated with rates in the first year overlapped with those associated with rates in subsequent years.

### RR of VTE among cancer patients compared with the general population

Overall, the risk of VTE was higher among cancer patients than in the general population, after adjustment for comorbid conditions (adjusted RR (aRR)=4.7, 95% CI=4.3–5.1) (Table 2). The aRR of VTE declined with increasing age at diagnosis, particularly for events during the first year after cancer diagnosis (aRR=21.0, 95% CI=11.0–39.9 among those aged <50 years *vs* aRR=7.0, 95% CI=1.7–29.6 among those aged at least 90 years) (*P*=0.14) (Supplementary Table 2). The aRR of VTE varied by cancer site, with higher RRs for oesophagus, pancreas and brain cancers or multiple myeloma and lower RRs for breast, endometrial and kidney cancer. For most cancer sites, the aRRs of VTE were higher during the first and second years of follow-up than in subsequent years. Surgery within 90 days of VTE conferred a significantly increased risk of VTE (see Supplementary Table 5). This was true for all years of follow-up.

### Cancer stage, treatment, site and risk of VTE

Our sub-analysis of patients with cancer records in both the DCR and DNRP included 40 994 cancer patients diagnosed between 1997 and 2003 (comprising 91.3% of cases identified in the DNRP during this period) and their 204 970 matched cancer-free members of the general-population. The effect of cancer on VTE risk increased with advancing tumour stage (aRR (95% CI)=2.9 (1.5–5.5); 2.9 (2.4–3.5); 7.5 (6.0–9.4); and 17.1 (12.6–23.3) among patients with stage I, II, III and IV disease, respectively, Table 3).

VTE IRs were highest among patients who received initial treatment of either chemotherapy alone or no/symptomatic treatment compared with patients treated with any other regimen or combination therapy (Table 3). After adjusting for comorbidity, age and sex, relative to the general-population cohort, VTE risk in cancer patients was strongest in those treated with any chemotherapy-containing regimen as part of initial cancer treatment (aRR=18.5, 95% CI=11.9–28.7 for chemotherapy alone and aRR=16.2, 95% CI=12.0–21.7 for chemotherapy combined with other treatments). VTE risk among patients who received chemotherapy within 4 months of cancer diagnosis remained substantially elevated during the first 2 years after cancer diagnosis, whereas it diminished substantially after the first year among patients treated with radiotherapy or surgery (Supplementary Table 3).

In the cancer cohort only, we examined the RR of VTE for tumour site, stage and treatment, while adjusting for sex, age, county and comorbid conditions. Compared with colon cancer, VTE risk was higher for brain, liver, ovary and pancreas cancers and lower for breast cancer and melanoma after controlling for stage and treatment (Supplementary Table 4). Likewise, chemotherapy was associated with a higher VTE risk compared with no/symptomatic treatment. VTE risk increased markedly with advancing stage.

## Discussion

We found that cancer patients had a greater risk for hospitalisation with VTE (1.8%) than cancer-free members of the general population (0.8%). The overall incidence of VTE in the cancer cohort is consistent with that reported in other studies (1.2% within the first 6 months; 1.6% within the first 2 years and 2.0% over all years of follow-up after cancer diagnosis (Blom *et al*, 2006a; Chew *et al*, 2006; Stein *et al*, 2006). VTE risk was increased over eight-fold during the first year following cancer diagnosis, over three-fold during the second year and over two-fold during subsequent years. In addition to survival time, strong predictors of VTE were cancer site, stage and type of initial cancer treatment.

The cancers we found associated with especially high rates of VTE (pancreas, liver, brain and multiple myeloma) are consistent with other research (Baron *et al*, 1998; Levitan *et al*, 1999; Blom *et al*, 2005; Blom *et al*, 2006b; Chew *et al*, 2006). Pancreas cancer has been associated with a high VTE risk (Chew *et al*, 2006; Ogren *et al*, 2006). Although it is frequently metastatic at diagnosis and may be associated with VTE on that basis alone, it has been suggested that an unknown VTE risk factor inherent to pancreas cancer may further increase risk (Ogren *et al*, 2006).

An important finding of our study is the high VTE risk associated with multiple myeloma, consistent with some published findings (Blom *et al*, 2006b; Khorana *et al*, 2007). New treatments for myeloma emerged during the period of our analysis, including the anti-angiogenic agents thalidomide and lenalidomide (Hales, 1999; Singhal *et al*, 1999). Recent reports suggest that thromboprophylaxis in myeloma patients may decrease the risk of VTE associated with these treatments (Knight *et al*, 2006; Falanga and Marchetti, 2009).

We confirmed the findings from Keenan and White, who concluded no evidence of a sex difference in VTE incidence either during hospitalisation or in the first year following cancer diagnosis (Keenan and White, 2007). Similar to our findings, studies show a decline in the overall IR of VTE in cancer patients with longer follow-up (Blom *et al*, 2005; Chew *et al*, 2006; White *et al*, 2007). Despite this, the excess risk of VTE in the cancer cohort compared with the general population prevailed throughout follow-up possibly because of patient, cancer and treatment related factors. A study of ovarian cancer patients suggested that early thrombotic events were associated with cancer treatment, whereas later events correlated with older age, a history of thrombosis, advanced stage and residual disease (Rodriguez *et al*, 2007).

The greater overall excess risk of VTE among cancer patients with advanced stage in our study agrees with other studies (Blom *et al*, 2006b; Chew *et al*, 2006; Rodriguez *et al*, 2007), and was evident even after adjusting for cancer site. Furthermore, our findings clearly showed that chemotherapy is a predictor of VTE in cancer patients, as has been reported (Otten *et al*, 2004; Blom *et al*, 2006b; Khorana *et al*, 2007). This excess risk was evident even after adjusting for cancer site and stage.

Regarding surgery, White *et al.* (2007) reported that patients surgically treated for cancers of the colon, breast and ovary had the lowest VTE incidence within 3 months of diagnosis compared with patients with cancer at other sites, whereas those with gliomas had the highest incidence in that time period. Although elevated relative to the general population, in our study the IR associated with surgery was not as high as that for chemotherapy. However, surgery is not a treatment option for all cancers (e.g., haematological cancers or those metastatic at diagnosis). Surgical patients may also have received post-surgical thromboprophylaxis (White *et al*, 2007), may have been selected for better performance status and overall health status, and/or may have had non-advanced (thus operable) disease at diagnosis. If true, these factors would decrease the apparent VTE risk among surgical patients.

Recent surgery is a strong transient risk factor for VTE, denoted ‘provoked VTE’ (Glynn and Rosner, 2005; Huerta *et al*, 2007). Our findings regarding such ‘provoked VTE’ concur with those of Huerta and colleagues (Huerta *et al*, 2007), who reported a nine-fold excess risk of VTE among individuals who had surgery up to 6 months before VTE diagnosis.

Strengths of our study include prospectively collected data and complete follow-up, reducing selection bias. Cancer diagnoses recorded in the DCR and DNRP have a high validity (Storm, 1988). We had a large sample size, enabling the study of many cancers, including rare cancers, such as multiple myeloma. Inability to examine rare cancers has been a limitation of other, smaller, studies (Heit *et al*, 2001; Blom *et al*, 2005).

Limitations of our study include lack of clinical characteristics and personal detail regarding the subjects. In particular, our findings may have been affected by unmeasured confounding by VTE risk factors, such as post-menopausal hormone replacement therapy and thromboprophylaxis, which could contribute to or diminish the observed cancer effect on risk of VTE. We relied on recorded registry diagnoses, which are not perfect. VTE diagnosis in the DNRP has an estimated positive predictive value of 75% (95% CI=71.9–77.9%) (Severinsen *et al*, 2010). Our outcome variable, VTE, includes upper extremity VTE, which has no dedicated ICD code (Sundhedsstyrelsen, 1993) and can occur as a complication of indwelling catheters in cancer patients (Bernardi *et al*, 2001). However, most patients have VTE at sites other than the upper extremities (Arcelus *et al*, 2003). Our inclusion of superficial venous thrombosis may have contributed to the elevated IRs associated with cancer treatment and in the first year after cancer diagnosis because superficial venous thrombosis may result from venous catheters associated with chemotherapy or surgery. Cancer patients may have received heightened surveillance for VTE, leading to surveillance bias and inflating our VTE RR estimates. Such bias is unlikely to extend beyond 1 year of follow-up; when most cancer patients receive active treatment and close medical observation (Rodriguez *et al*, 2007).

The DCR records treatment administered within 4 months of diagnosis. Therefore, if a treatment increases VTE risk and is administered over 4 months after cancer diagnosis, we may have underestimated its impact. A ‘watchful waiting’ strategy (where treatment was administered on appearance of symptoms) may explain the consistently high excess risk of VTE among prostate cancer patients throughout follow-up, whereas VTE risk associated with many other cancers declined over time.

Our study furthers the understanding of the association between cancer and VTE. Within the cancer cohort, the elevated risk of VTE for some cancer sites, even after adjusting for cancer treatment, stage, age, sex and potential confounding diseases, suggests that increased VTE occurrence is an inherent biological property of some tumours (e.g., brain and pancreas cancers).

In cancers associated with a slightly elevated risk of VTE compared with the general population (e.g., breast cancer), VTE may be attributable to cancer-directed treatment or stage (Stein *et al*, 2006; Hernandez *et al*, 2009). VTE may also be related to the biological aggressiveness of the malignant process in general as suggested by the elevated risk of VTE in all patients with advanced stage. However, cancer patients are also likely to be burdened with increased medical intervention and forced sedentary lifestyle, factors that would increase the VTE risk. Nonetheless, in our study, even patients with early-stage cancer had increased VTE risk relative to the general population. The increased risk persisted among patients who received treatments other than chemotherapy or no/symptomatic treatment and remained elevated throughout follow-up. These findings suggest that some factors underlying the association between VTE and cancer are present even at the earliest stages of disease. The likely multi-factorial mechanism for increased VTE risk in cancer patients remains to be elucidated (Kakkar, 2010).

## Figures and Tables

**Table 1:** Characteristics of the cancer and general-population cohorts and the distribution of incident hospitalisation for VTE (Danish National Registry of Patients, 1997–2005)

	**Cancer patients**			**General-population cohort**		
**Characteristic**	**Total number**	**VTE number (%)** a	**Observation time, person-years**	**Total number**	**VTE number (%)** a	**Observation time, person-years**
Overall	57 591	1023 (1.8%)	127 492	287 476	2204 (0.8%)	1 087 946
						
*Sex*						
Female	30 060	527 (1.8%)	74 825	150 078	1088 (0.7%)	592 092
Male	27 531	496(1.8%)	52 667	137 398	1116 (0.8%)	495 854
						
*Age at diagnosis, years*						
<50	7356	105 (1.4%)	24 427	36 792	82 (0.2%)	158 635
50–59	10 262	215 (2.1%)	27 337	51 231	204 (0.4%)	215 803
60–69	14 231	305 (2.1%)	31 885	71 143	502 (0.7%)	285 230
70–79	16 068	271 (1.7%)	30 405	80 181	882 (1.1%)	291 450
80–89	8702	119 (1.4%)	12 504	43 245	497 (1.1%)	126 419
90+	972	8 (0.8%)	935	4884	37 (0.8%)	10 409
						
*Cancer site*						
Oesophagus	938	14 (1.5%)	872	4682	29 (0.6%)	17 155
Stomach	1172	18 (1.5%)	1417	5851	61 (1.0%)	21 933
Colon	5595	126 (2.3%)	13 252	27 922	230 (0.8%)	100 694
Rectum	2778	55 (2.0%)	7361	13 866	113 (0.8%)	52 623
Liver	550	6 (1.1%)	295	2746	11 (0.4%)	10 216
Pancreas	1671	36 (2.2%)	881	8342	80 (1.0%)	30 467
Lung	7975	127 (1.6%)	7872	39 810	336 (0.8%)	151 350
Breast	8586	119 (1.4%)	30 391	42 869	234 (0.5%)	171 760
Cervix	1019	16 (1.6%)	3499	5090	24 (0.5%)	22 341
Endometrium	1453	22 (1.5%)	5049	7258	59 (0.8%)	28 604
Ovary	1534	49 (3.2%)	4066	7663	48 (0.6%)	31 828
Prostate	4457	98 (2.2%)	9757	22 230	219 (1.0%)	70 899
Kidney	1376	12 (0.9%)	2972	6864	37 (0.5%)	25 538
Urinary bladder	2445	62 (2.5%)	5980	12 205	116 (1.0%)	45 436
Brain	1133	19 (1.7%)	1071	5653	37 (0.7%)	21 685
Hodgkin lymphoma	336	6 (1.8%)	1143	1680	4 (0.2%)	6851
Non-Hodgkin lymphoma	2003	47 (2.3%)	4788	9999	79 (0.8%)	38 013
Leukaemia	1516	41 (2.7%)	2943	7567	66 (0.9%)	28 060
Multiple myeloma	643	26 (4.0%)	1149	3211	30 (0.9%)	11 972
Bone	229	4 (1.4%)	541	1143	7 (0.6%)	4709

Abbreviation: VTE=venous thromboembolism.

a VTEs which occurred in the year before cancer diagnosis/ index date were excluded.

**Table 2:** IRs of hospitalisation for VTE per 1000 person-years in the cancer cohort

**Characteristic**	**IR (95% CI)**	**aRR (95% CI)**
Overall	8.0 (7.6–8.5)	4.7 (4.3–5.1)
		
*Sex*		
Female	7.0 (6.5–7.7)	4.8 (4.2–5.4)
Male	9.4 (8.6–10.3)	4.6 (4.1–5.3)
		
*Age, years*		
<50	4.3 (3.6–5.2)	8.7 (6.2–12.2)
50–59	7.9 (6.9–9.0)	9.6 (7.6–12.2)
60–69	9.6 (8.6–10.7)	5.6 (4.7–6.6)
70–79	8.9 (7.9–10.0)	3.1 (2.7–3.7)
80–89	9.5 (8.0–11.4)	2.9 (2.3–3.7)
90+	8.6 (4.2–17.1)	3.0 (1.1–8.7)
		
*Cancer site*		
Oesophagus	16.1 (9.5–27.1)	11.6 (3.8–35.0)
Stomach	12.7 (8.0–20.2)	8.9 (3.8–20.7)
Colon	9.5 (8.0–11.3)	4.8 (3.7–6.2)
Rectum	7.5 (5.7–9.7)	4.0 (2.8–5.9)
Liver	20.4 (9.2–45.3)	—a
Pancreas	40.9 (29.5–56.7)	16.3 (8.1–32.6)
Lung	16.1 (13.6–19.2)	8.0 (6.0–10.7)
Breast	3.9 (3.3–4.7)	3.3 (2.6–4.2)
Cervix	4.6 (2.8–7.5)	10.8 (4.2–28.1)
Endometrium	4.4 (2.9–6.6)	2.2 (1.1–3.9)
Ovary	12.1 (9.1–15.9)	10.1 (6.1–16.7)
Prostate	10.0 (8.2–12.2)	3.1 (2.4–4.1)
Kidney	4.0 (2.3–7.1)	2.7 (1.1–6.6)
Urinary bladder	10.4 (8.1–13.3)	4.5 (3.1–6.4)
Brain	17.7 (11.3–27.8)	19.8 (7.1–55.2)
Hodgkin Lymphoma	5.3 (2.4–11.7)	9.7 (2.3–41.3)
Non-Hodgkin Lymphoma	9.8 (7.4–13.1)	6.6 (4.2–10.5)
Leukaemia	13.9 (10.3–18.9)	9.1 (5.3–15.8)
Multiple Myeloma	22.6 (15.4–33.22)	46.1 (13.1–162.0)
Bone	7.4 (2.8–19.7)	9.7 (0.7–130.9)

Abbreviations: aRR=adjusted relative risk; CI=confidence interval; IR=incidence rate; VTE=venous thromboembolism.

Cox proportional hazards regression models computing the adjusted relative risks (aRRs) (Adjusted for myocardial infarction, congestive heart failure, peripheral vascular disease, chronic obstructive pulmonary disease, inflammatory bowel disease, peptic ulcer disease, liver disease, renal disease, diabetes, obesity, acute pancreatitis, alcoholism and hypertension when the number of VTE events for a given comorbidity was sufficient) of hospitalisation for VTE in the cancer cohort compared with the general-population (Danish National Registry of Patients, 1997–2005). *Please see Supplementary Information for IR and RR by time since diagnosis/index date.*

a Too few VTE events to estimate incidence.

**Table 3:** IRs of hospitalisation for VTE^a^ per 1000 person-years in the cancer cohort (*n*=40 994) and aRRs^b^ of hospitalisation for venous thromboembolism in the cancer cohort compared with the general-population (*n*=204 970) (DCR, 1997–2003)^c^

**Characteristic**	***N***	**IR (95% CI)**	**aRR**^b^ **(95% CI)**
*Cancer stage* c			
Stage I	1240	44 (2.7–7.1)	2.9 (1.5–5.5)
Stage II	14520	44.9 (4.0–5.7)	2.9 (2.4–3.5)
Stage III	10499	11.1 (9.7–12.7)	7.5 (6.0–9.4)
Stage IV	9125	27.7 (243.0–32.0)	17.1 (12.6–23.3)
Unspecified	5610	12.2 (10.1–14.8)	5.6 (4.1–7.5)
			
*Treatment* c,d			
No/symptomatic	8565	20.8 (17.3–25.0)	8.4 (6.2–11.4)
Chemotherapy only	3026	23.1 (19.0–28.1)	18.5 (11.9–28.7)
Radiation only	2512	10.1 (7.2–14.1)	8.9 (5.0–16.0)
Surgery only	16564	6.5 (5.7–7.3)	3.2 (2.7- 3.8)
Other^e^	781	13.4 (7.6–23.7)	6.0 (2.3–15.6)
Combination therapy	8625	8.5 (7.3–9.9)	8.6 (6.7–11.1)
Unspecified	921	9.2 (4.8–17.6)	5.8 (2.1–16.6)
			
*Treatment including* c			
No/symptomatic	8565	20.8 (17.3–25.0)	8.4 (6.2–11.4)
Chemotherapy	7154	14.0 (12.2–16.2)	16.2 (12.0–21.7)
Radiation	6943	8.2 (6.8–9.9)	7.9 (5.8–10.7)
Surgery	24525	7.0 (6.3–7.7)	4.1 (3.6- 4.7)
Other^e^	781	13.4 (7.6–23.7)	6.0 (2.3–15.6)
Unspecified	921	9.2 (4.8–17.6)	5.8 (2.1–16.6)

Abbreviations: aRR=adjusted relative risk; CI=confidence interval; DCR=Danish Cancer Registry; IR=incidence rate; VTE=venous thromboembolism.

Please see Supplementary Information for IR and RR by time since diagnosis/index date.

a We excluded VTEs that occurred in the year before diagnosis/ index date.

b Adjusted for age, sex, myocardial infarction, congestive heart failure, peripheral vascular disease, chronic obstructive pulmonary disease, inflammatory bowel disease, peptic ulcer disease, liver disease, renal disease, diabetes, obesity, acute pancreatitis, alcoholism and hypertension when the number of VTE events for a given comorbidity was sufficient.

c To obtain data on cancer stage and treatment, analyses are based on cancer patients in the DCR and their matched members of the general-population cohort.

d Mutually–exclusive treatment categories

e Others describe treatment other than chemotherapy, radiation and/or surgery. This includes cryocoagulation, anti-hormone therapy and other treatments not further specified.
