# Supplementary material for: Hospitalisation for venous thromboembolism in cancer patients and the general population: a population-based cohort study in Denmark, 1997–2006
Source: Br J Cancer. 2010 Sep 14;103(7):947–53. doi: 10.1038/sj.bjc.6605883 (PMC2965880; doi:10.1038/sj.bjc.6605883)
Supplement: Supplementary Tables 1–5 [file 6605883x1.doc]

**ON-LINE SUPPLEMENTARY TABLES**

**Supplementary Table 2. Incidence rates (IRs) of hospitalization for venous thromboembolism (VTE) per 1,000 person-years in the cancer cohort. Cox proportional hazards regression models to compute adjusted relative risks (aRRs)^†^ of hospitalization for venous thromboembolism in the cancer cohort compared to the general-population** (Danish National Registry of Patients, 1997-2005)

| **Characteristic** | | **Years since cancer diagnosis** | | | | **Years since cancer diagnosis/index date** | | | |
| --- | --- | --- | --- | --- | --- | --- | --- | --- | --- |
|  |  | **Overall**  **IR (95%CI)** | **<1 year**  **IR (95%CI)** | **1≤ years <2**  **IR (95%CI)** | **2+ years**  **IR (95%CI)** | **Overall**  **aRR (95%CI)** | **<1 year**  **aRR (95% CI)** | **1≤ years <2**  **aRR (95%CI)** | **2+ years**  **aRR (95%CI)** |
| **Overall** | | 8.0 ( 7.6- 8.5) | 15.0 (13.8-16.2) | 6.3 (5.4- 7.3) | 4.2 (3.7- 4.7) | 4.7 (4.3- 5.1) | 8.6 ( 7.6- 9.9) | 3.6 (2.9- 4.4) | 2.4 (2.1- 2.9) |
| **Sex^1^** | |  |  |  |  |  |  |  |  |
|  | Female | 7.0 ( 6.5- 7.7) | 13.8 (12.3-15.4) | 5.8 (4.7- 7.2) | 3.6 (3.1- 4.3) | 4.8 (4.2- 5.4) | 9.3 ( 7.7-11.3) | 4.3 (3.2- 5.8) | 2.4 (1.9- 3.0) |
|  | Male | 9.4 ( 8.6-10.3) | 16.4 (14.7-18.4) | 6.9 (5.5- 8.7) | 5.1 (4.3- 6.1) | 4.6 (4.1- 5.3) | 8.18 ( 6.78- 9.87) | 3.1 (2.3- 4.2) | 2.6 (2.0- 3.3) |
| **Age, years** | |  |  |  |  |  |  |  |  |
|  | <50 | 4.3 ( 3.6- 5.2) | 9.1 (7.1-11.9) | 4.2 (2.7- 6.5) | 2.1 (1.4- 3.0) | 8.7 (6.2-12.2) | 21.0 (11.001-39.9) | 13.8 (5.5-34.9) | 3.5 (2.1- 5.9) |
|  | 50 – 59 | 7.9 ( 6.9- 9.0) | 15.8 (13.3-18.8) | 7.1 (5.2- 9.7) | 3.5 (2.7- 4.7) | 9.6 (7.6-12.2) | 16.5 (11.3-24.0) | 14.1 (7.2-27.5) | 3.9 (2.6- 5.9) |
|  | 60 – 69 | 9.6 ( 8.6-10.7) | 18.0 (15.6-20.8) | 7.0 (5.2- 9.3) | 5.0 (4.0- 6.3) | 5.6 (4.7- 6.6) | 12.0 ( 9.1-15.9) | 5.1 (3.2- 7.9) | 2.5 (1.8- 3.3) |
|  | 70 – 79 | 8.9 ( 7.9-10.0) | 14.9 (12.7-17.4) | 6.1 (4.4- 8.3) | 5.7 (4.6- 7.2) | 3.1 (2.7- 3.7) | 6.2 ( 4.9- 8.0) | 1.7 (1.2- 2.6) | 1.9 (1.5- 2.6) |
|  | 80 – 89 | 9.5 ( 8.0-11.4) | 14.9 (11.9-18.7) | 7.2 (4.6-11.1) | 5.3 (3.6- 7.8) | 2.9 (2.3- 3.7) | 4.3 ( 3.1- 6.0) | 2.1 (1.2- 3.7) | 1.8 (1.0- 3.0) |
|  | 90+ | 8.6 ( 4.3-17.1) | 15.7 (7.5-33.0) | 4.7 (0.7-33.2) | 0.00 ( . - . ) | 3.0 (1.1- 8.7) | 7.0 ( 1.7-29.6) | 1.7 (0.2-18.2) | * |
| **Cancer site** | |  |  |  |  |  |  |  |  |
|  | Oesophagus | 16.1 ( 9.5-27.1) | 22.9 (12.7-41.3) | 12.5 (3.1-49.9) | 4.3 (0.6-30.8) | 11.6 (3.8-35.0) | 41.9 ( 5.2-335.4) | 1.6 (0.1-17.8) | 3.9 (0.2-63.3) |
|  | Stomach | 12.7 ( 8.0-20.2) | 18.1 (10.0-32.7) | 11.0 (3.5-34.0) | 7.5 (2.8-19.9) | 8.9 (3.8-20.7) | 10.6 (3.2-34.6) | * | 10.7 (1.1-102.7) |
|  | Colon | 9.5 ( 8.0-11.3) | 19.3 (15.5-24.0) | 6.8 (4.3-10.6) | 4.4 (3.1- 6.4) | 4.8 (3.7- 6.2) | 10.2 (6.8-15.2) | 3.7 (1.9- 7.2) | 1.9 (1.2- 3.1) |
|  | Rectum | 7.5 (5.7- 9.7) | 13.5 (9.5-19.4) | 5.0 (2.5-10.0) | 4.8 (3.0- 7.7) | 4.0 (2.8- 5.9) | 8.7 (4.6-16.3) | 2.7 (1.1- 6.6) | 2.2 (1.2- 4.2) |
|  | Liver | 20.4 ( 9.2-45.3) | 22.6 (8.5-60.1) | 18.0 (2.5-127.7) | 16.2 (2.3-115.2) | * | * | * | * |
|  | Pancreas | 40.9 (29.5-56.7) | 56.1 (39.9-78.8) | 24.4 (7.9-75.8) | 0.0 (----)* | 16.3 (8.1-32.6) | 16.2 (7.7-34.3) | * | * |
|  | Lung | 16.1 (13.6-19.2) | 22.7 (18.4-27.9) | 14.1 (9.2-21.7) | 7.2 (4.5-11.4) | 8.0 (6.0-10.7) | 10.6 (7.2-15.5) | 7.3 (3.3-16.1) | 3.5 (1.6- 7.7) |
|  | Breast | 3.9 (3.3- 4.7) | 6.9 (5.3- 9.0) | 2.9 (1.8- 4.5) | 2.9 (2.2- 3.9) | 3.3 (2.6- 4.2) | 9.2 (5.7-14.9) | 3.0 (1.6- 5.5) | 2.2 (1.5- 3.1) |
|  | Cervix | 4.6 (2.8- 7.5) | 5.7 (2.4-13.8) | 5.9 (2.2-15.6) | 3.6 (1.7- 7.6) | 10.8 (4.2-28.1) | 8.9 (1.6-48.9) | 15.9 (1.8-143.1) | 4.9 (1.2-19.4) |
|  | Endometrium | 4.4 (2.9- 6.6) | 8.8 (4.9-15.8) | 4.0 (1.5-10.7) | 2.5 (1.2- 5.3) | 2.2 (1.2- 3.9) | 4.2 (1.5-12.1) | 2.4 (0.7- 9.1) | 1.5 (0.5- 4.2) |
|  | Ovary | 12.1 (9.1-15.9) | 22.3 (15.3-32.6) | 16.4 (9.7-27.7) | 4.0 (2.0- 8.0) | 10.1 (6.1-16.7) | 16.4 (7.2-37.5) | 31.6 (7.2-139.1) | 4.7 (1.7-13.3) |
|  | Prostate | 10.0 (8.2-12.2) | 9.8 (7.0-13.6) | 8.1 (5.2-12.8) | 11.4 (8.5-15.4) | 3.1 (2.4- 4.1) | 3.6 (2.3- 5.8) | 2.06 ( 1.17- 3.60) | 4.0 (2.6- 6.2) |
|  | Kidney | 4.0 (2.3- 7.1) | 6.3 (2.8-14.1) | 3.2 (0.8-12.8) | 2.9 (1.1- 7.6) | 2.7 (1.1- 6.6) | 3.8 (1.1-13.2) | 8.6 (0.8-95.5) | 1.7 (0.2-18.2) |
|  | Urinary bladder | 10.4 (8.1-13.3) | 18.1 (12.9-25.3) | 12.2 (7.3-20.2) | 4.5 (2.6- 7.8) | 4.5 (3.1- 6.4) | 8.0 (4.5-14.3) | 5.6 (2.4-13.4) | 3.1 (1.4- 6.5) |
|  | Brain | 17.7 (11.3-27.8) | 28.1 (17.5-45.2) | 0.0 (----)* | 7.1 (1.8-28.6) | 19.8 (7.1-55.2) | 81.7 (10.7-623.3) | * | 1.4 (0.1-13.3) |
|  | HL | 5.3 (2.4-11.7) | 17.5 (7.3-42.1) | 0.0 (----)* | 1.6 (0.2-11.4) | 9.7 (2.3-41.3) | 31.7 (3.0-340.8) | * | 4.0 (0.3-64.0) |
|  | NHL | 9.8 (7.4-13.1) | 21.5 (15.2-30.4) | 6.0 (2.7-13.4) | 3.9 (2.0- 7.5) | 6.6 (4.2-10.5) | 20.7 (8.9-48.0) | 2.2 (0.7- 7.0) | 3.0 (1.1- 7.6 |
|  | Leukaemia | 13.9 (10.3-18.9) | 21.0 (13.7-32.3) | 16.6 (9.2-30.0) | 7.0 (3.7-13.5) | 9.1 (5.3-15.8) | 20.3 (7.3-56.4) | 7.9 (2.4-26.3) | 3.6 (1.3-10.1) |
|  | MM | 22.6 (15.4-33.2) | 41.2 (26.3-64.5) | 3.6 (0.5-25.8) | 14.6 (6.5-32.4) | 46.1 (13.1-162.0) | * | * | 13.8 (2.8-68.6) |
|  | Bone | 7.4 ( 2.8-19.7) | 12.3 ( 3.1-49.0) | 19.3 ( 4.8-77.1) | * | 9.7 (0.7 – 130.9) | * | * | * |

CI: confidence interval, HL: Hodgkin lymphoma; MM: multiple myeloma; NHL: non-Hodgkin lymphoma

*No or too few venous thromboembolism events to estimate incidence.

^†^Adjusted for myocardial infarction, congestive heart failure, peripheral vascular disease, chronic obstructive pulmonary disease, inflammatory bowel disease, peptic ulcer disease, liver disease, renal disease, diabetes, obesity, acute pancreatitis, alcoholism, and hypertension when the number of VTE events for a given comorbidity was sufficient.

^‡^Crude estimates presented (not adjusted for any of the comorbidities listed above) because of the limited number of VTE events.

**^1^** Estimates for all cancers including sex-specific cancers.

**Supplementary Table 3. Incidence rates (IRs) of hospitalization for venous thromboembolism (VTE) per 1,000 person-years in the cancer cohort (n=40,994) and adjusted relative risks (aRRs)^†^ of hospitalization for venous thromboembolism in the cancer cohort compared to the general-population (n=204,970) (Danish Cancer Registry, 1997-2003)^‡^**

| **Characteristic** | | **N** | **Years since cancer diagnosis** | | | | **Years since cancer diagnosis/index date** | | | |
| --- | --- | --- | --- | --- | --- | --- | --- | --- | --- | --- |
|  |  |  | **Overall**  **IR (95%CI)** | **<1 year**  **IR (95%CI)** | **1≤ years <2**  **IR (95%CI)** | **2+ years**  **IR (95%CI)** | **Overall**  **aRR (95%CI)** | **<1 year**  **aRR (95%CI)** | **1≤ years <2**  **aRR (95%CI)** | **2+ years**  **aRR (95%CI)** |
| **Cancer stage** | |  |  |  |  |  |  |  |  |  |
|  | Stage I | 1240 | 4.4 (2.7- 7.1) | 10.8 (6.2-19.1) | 2.2 (0.6- 8.8) | 1.6 (0.5- 5.0) | 2.9 (1.5-5.5) | 6.5 (2.4-18.1) | 2.9 (0.5-17.5) | 1.3 (0.3- 5.2) |
|  | Stage II | 14520 | 4.98 (4.2- 5.7) | 7.9 (6.5- 9.7) | 4.6 (3.4- 6.3) | 2.8 (2.1- 3.7) | 2.9 (2.4- 3.5) | 4.5 (3.4- 5.9) | 2.7 (1.8- 4.1) | 1.6 (1.1- 2.4) |
|  | Stage III | 10499 | 11.1 (9.7-12.7) | 17.6 (14.8-20.8) | 8.2 (6.0-11.3) | 5.8 (4.2- 7.9) | 7.5 (6.0- 9.4) | 12.6 (9.0-17.5) | 6.4 (3.9-10.8) | 3.3 (2.1- 5.2) |
|  | Stage IV | 9125 | 27.7 (24.0-32.0) | 40.7 (34.9-47.4) | 9.6 (5.6-16.5) | 7.4 (4.0-13.7) | 17.1 (12.6-23.3) | 25.1 (17.1-36.9) | 6.4 (2.5-16.0) | 5.9 (1.9-18.6) |
|  | Unspecified | 5610 | 12.2 (10.1-14.8) | 19.4 (15.3-24.7) | 10.5 (6.9-16.2) | 5.6 (3.5- 8.9) | 5.6 (4.1- 7.5) | 8.6 (5.6-13.0) | 4.5 (2.2- 9.2) | 2.9 (1.5- 5.6) |
| **Treatment**¤ | |  |  |  |  |  |  |  |  |  |
|  | No/symptomatic | 8565 | 20.8 (17.3-25.0) | 31.3 (25.6-38.3) | 9.4 (5.0-17.4) | 6.1 (3.0-12.1) | 8.4 (6.2-11.4) | 12.1 (8.3-17.7) | 3.4 (1.0-12.0) | 2.2 (0.8- 5.8) |
|  | Chemotherapy only | 3026 | 23.1 (19.0-28.1) | 39.0 (31.3-48.6) | 12.4 (7.0-21.8) | 6.7 (3.5-12.8) | 18.5 (11.9-28.7) | 32.4 (17.1-61.4) | 10.5 (3.5-31.4) | 7.1 (2.1-24.3) |
|  | Radiation only | 2512 | 10.1 (7.2-14.1) | 19.2 (13.4-27.6) | 4.3 (1.4-13.2) | 1.7 (0.4- 6.9) | 8.9 (5.0-16.0) | 21.4 (8.6-53.5) | 3.8 (0.7-19.6) | 1.3 (0.2- 7.0) |
|  | Surgery only | 16564 | 6.5 (5.7- 7.3) | 11.3 (9.7-13.3) | 5.2 (3.9- 6.8) | 3.4 (2.6- 4.4) | 3.2 (2.7- 3.8) | 6.0 (4.7- 7.7) | 2.4 (1.7- 3.5) | 1.6 (1.1- 2.2) |
|  | Other^§^ | 781 | 13.4 (7.6-23.7) | 19.8 (10.3-38.1) | 4.4 (0.6-31.1) | 9.5 (2.4-38.1) | 6.0 (2.3-15.6) | 8.0 (2.3-27.9) | 0.00 ( 0.00- . ) | 5.4 (0.5-64.1) |
|  | Combination therapy | 8625 | 8.5 (7.3- 9.9) | 13.7 (11.3-16.7) | 7.4 (5.4-10.3) | 4.3 (3.1- 6.0) | 8.6 (6.7-11.1) | 12.4 (8.5-18.1) | 12.9 (6.7-25.1) | 5.0 (2.9- 8.4) |
|  | Unspecified | 921 | 9.2 (4.8-17.6) | 10.8 (4.5-26.0) | 9.5 (2.4-38.1) | 6.4 (1.6-25.8) | 5.8 (2.1-16.6) | 7.8 (1.7-35.4) | 3.9 (0.2-63.3) | 3.6 (0.5-26.1) |
| **Treatment including:** | |  |  |  |  |  |  |  |  |  |
|  | No/symptomatic | 8565 | 20.8 (17.3-25.0) | 31.3 (25.6-38.3) | 9.4 (5.0-17.4) | 6.1 (3.0-12.1) | 8.4 (6.2-11.4) | 12.1 (8.3-17.7) | 3.4 (1.0-12.0) | 2.2 (0.8- 5.8) |
|  | Chemotherapy | 7154 | 14.0 (12.2-16.2) | 25.2 (21.3-29.7) | 9.7 (6.9-13.8) | 4.1 (2.7- 6.4) | 16.2 (12.0-21.7) | 24.1 (15.9-36.5) | 17.0 (7.9-36.7) | 7.1 (3.3-15.3) |
|  | Radiation | 6943 | 8.2 (6.8- 9.9) | 14.4 (11.5-18.1) | 5.9 (3.8- 9.3) | 3.7 (2.4- 5.7) | 7.9 (5.8-10.7) | 15.0 (9.3-24.3) | 8.6 (3.8-19.4) | 3.2 (1.7- 5.9) |
|  | Surgery | 24525 | 7.0 (6.3- 7.7) | 11.7 (10.3-13.3) | 6.0 (4.9- 7.5) | 3.6 (2.9- 4.4) | 4.1 (3.6- 4.7) | 6.9 (5.6- 8.4) | 3.8 (2.8- 5.1) | 2.0 (1.5- 2.6) |
|  | Other^§^ | 781 | 13.4 (7.6-23.7) | 19.8 (10.3-38.1) | 4.4 (0.6-31.1) | 9.5 (2.4-38.1) | 6.0 (2.3-15.6) | 8.0 (2.3-27.9) | 0.00 ( 0.00- . ) | 5.4 (0.5-64.1) |
|  | Unspecified | 921 | 9.2 (4.8-17.6) | 10.8 (4.5-26.0) | 9.5 (2.4-38.1) | 6.4 (1.6-25.8) | 5.8 (2.1-16.6) | 7.8 (1.7-35.4) | 3.9 (0.2-63.3) | 3.6 (0.5-26.1) |

CI: confidence interval

*No or too few venous thromboembolism events to estimate.

^†^Adjusted for age, sex, myocardial infarction, congestive heart failure, peripheral vascular disease, chronic obstructive pulmonary disease, inflammatory bowel disease, peptic ulcer disease, liver disease, renal disease, diabetes, obesity, acute pancreatitis, alcoholism, and hypertension when the number of VTE events for a given comorbidity was sufficient.

^‡^To obtain data on cancer stage and treatment, analyses are based on cancer patients in the Danish Cancer Registry (n = 40,994) (a subset of the cancer patients in the Danish National Registry of Patients) and their matched members of the general-population cohort (n = 204,970).

¤Mutually-exclusive treatment categories

^§^Other describes those who received treatment other than chemotherapy, radiation, and/or surgery. This includes cryocoagulation, anti-hormone therapy, and some other treatments not further specified.

**S**upplementary Table 4. Adjusted relative risks (aRRs) of hospitalization for venous thromboembolism in the cancer cohort (n=40,994) according to cancer site, stage and treatment after adjustment for age, sex, county of residence and comorbid conditions (Danish Cancer Registry, 1997-2003)

|  | **aRR** | **95%CI** |
| --- | --- | --- |
| **Site of cancer** |  |  |
| Colon | 1.0 | reference |
| Brain | 1.4 | 0.8 – 2.6 |
| Breast | 0.4 | 0.3 – 0.6 |
| Cervix uteri | 0.9 | 0.5 – 1.8 |
| Endometrium | 0.7 | 0.4 – 1.2 |
| Hodgkin lymphoma | 0.7 | 0.3 – 1.6 |
| Kidney | 0.7 | 0.4 – 1.3 |
| Leukaemia | 1.0 | 0.6 – 1.7 |
| Liver | 1.5 | 0.6 – 3.7 |
| Lung | 0.8 | 0.6 - 1.2 |
| Melanoma of the skin | 0.5 | 0.2 – 0.9 |
| Multiple myeloma | 1.2 | 0.7 – 2.2 |
| Non-Hodgkin lymphoma | 0.8 | 0.5 – 1.2 |
| Oesophagus | 0.7 | 0.3 – 1.5 |
| Ovary | 1.7 | 1.1 – 2.5 |
| Pancreas | 1.9 | 1.2 – 3.0 |
| Prostate | 1.0 | 0.7 – 1.5 |
| Rectum | 0.8 | 0.6 – 1.2 |
| Stomach | 0.6 | 0.3 – 1.3 |
| Urinary bladder | 1.4 | 0.9 – 2.0 |
| **Cancer-directed treatment** |  |  |
| None/ symptomatic | 1.0 | Reference |
| Chemotherapy | 1.5 | 1.1– 2.0 |
| Combination therapy | 0.9 | 0.7 – 1.2 |
| Other | 0.7 | 0.4 – 1.2 |
| Radiation | 1.0 | 0.6 – 1.4 |
| Surgery | 0.7 | 0.6 – 1.0 |
| Unspecified | 0.6 | 0.3 – 1.2 |
| **Cancer stage at diagnosis** |  |  |
| Stage I | 1.0 | Reference |
| Stage II | 1.2 | 0.7 – 2.1 |
| Stage III | 2.2 | 1.3 – 3.8 |
| Stage IV | 2.9 | 1.7 – 5.1 |
| Unspecified | 1.8 | 1.0 – 3.2 |

**Supplementary Table 5: Relative risk (RR) and associated 95% confidence intervals (95%CI) of VTE according to surgery 90 days before VTE. (Data from the Danish National Registry of Patients, 1997-2005, cancer cohort versus general population comparison cohort).**

|  | **Overall**  **RR (95% CI)** | **RR (95%CI)**  **<1 year** | **RR (95% CI)**  **1 to <=2 years** | **RR (95% CI)**  **2+ years** |
| --- | --- | --- | --- | --- |
| **Surgery <90 days before VTE** | | | | |
| **All** | 4.7 (4.3- 5.1) | 8.4 (7.3- 9.5) | 3.5 (2.8- 4.2) | 2.4 (2.1- 2.8) |
| **No** | 3.6 (3.3- 4.0) | 5.7 (4.9- 6.6) | 3.2 (2.6- 4.0) | 2.2 (1.9- 2.7) |
| **Yes** | * | * | * | * |
|  |  |  |  |  |
